# *KRAS*, *NRAS*, and *BRAF* Hot-Spot Mutations in Relation to Sidedness of Primary Colorectal Cancer: A Retrospective Cohort Study

**DOI:** 10.3390/diagnostics15020142

**Published:** 2025-01-09

**Authors:** Omer Abdelgadir, Yong-Fang Kuo, Anthony O. Okorodudu, M. Firoze Khan, Yu-Wei Cheng, Jianli Dong

**Affiliations:** 1 Graduate School of Biomedical Science, University of Texas Medical Branch, Galveston, TX 77555, USA; 2 School of Public and Population Health, University of Texas Medical Branch, Galveston, TX 77555, USA; yokuo@utmb.edu; 3 Department of Pathology, University of Texas Medical Branch, Galveston, TX 77555, USA; aookorod@utmb.edu (A.O.O.); mfkhan@utmb.edu (M.F.K.); 4 Department of Laboratory Medicine, Cleveland Clinic, Cleveland, OH 44195, USA

**Keywords:** *KRAS*, *NRAS*, *BRAF*, hot-spot mutation, colorectal cancer, tumor sidedness, molecular biomarker, tumor biomarker, pyrosequencing

## Abstract

**Background/Objective:** Studies have shown an association between colorectal cancer (CRC) sidedness and gene mutations that may affect CRC clinical behavior. This study examined the association between specific *KRAS*, *NRAS*, and *BRAF* hot-spot mutations and primary CRC sidedness. **Methods:** We performed a retrospective cohort analysis of 722 patients diagnosed with primary CRC and tested for *KRAS*, *NRAS*, and *BRAF* hot-spot mutations at the University of Texas Medical Branch (UTMB) from January 2016 through July 2023. Multivariable logistic regressions analyses were conducted. **Results:** *KRAS*, *NRAS*, and *BRAF* hot-spot mutations rates were 37.8%, 4.6%, and 6.1%, respectively. Right-sided primary CRC had the highest prevalence of mutated tumors (64%). *KRAS* and *BRAF* hot-spot mutations were significantly different according to tumor sidedness. *KRAS* p.Gly12Asp, p.Gly12Val, and p.Gly13Asp showed a significantly increased likelihood of right-sided primary CRC compared to *KRAS* wildtype, 128%, 134%, and 221% higher, respectively. Conversely, *KRAS* p.Gly12Val and p.Gly13Asp mutations were associated with decreased likelihood of rectal cancer (53% lower) and left-sided tumors (56% lower), respectively. *BRAF* p.Val600Glu mutation, as opposed to *BRAF* wildtype, was associated with a 278% higher likelihood of right-sided CRC. No significant associations were observed between *NRAS* mutations and primary CRC sidedness. **Conclusions:** In primary CRC, specific mutations in *KRAS* (p.Gly12Asp, p.Gly12Val, and p.Gly13Asp) and *BRAF* p.Val600Glu were associated with increased likelihood of right-sided tumors. *KRAS* p.Gly12Val and p.Gly13Asp mutations were associated with decreased likelihood of rectal cancer and left-sided tumors, respectively. These findings suggest that tumorigenesis and mutational processes differ based on tumor sidedness. Further studies are needed to substantiate these findings.

## 1. Introduction

There is growing attention to the role of CRC anatomical location. Studies suggest that CRC originating from the right-sided or left-sided colon are biologically different [1,2]. Beyond their developmental origins, these distinct anatomical regions within the colon and rectum harbor unique characteristics. They differ in bile acid concentrations, lymphatic drainage patterns, luminal environments, and mesenteric arteries blood supply [3,4,5].

Activating mutations in *KRAS*, *NRAS*, and *BRAF* proto-oncogenes are routinely evaluated in CRC to predict response to anti-EGFR monoclonal antibody therapy [6,7]. Cells in different anatomical locations of the colon and rectum can be influenced by distinct developmental programs, resulting in different gene expression and activities of cellular signaling pathways, with subsequent various vulnerabilities and tolerances to gene mutations [8]. In addition, cells in different locations of the colon and rectum may be exposed to unique carcinogens [9,10,11]. Together, the different intrinsic factors and external cellular environment may generate different spectrums of mutations in *KRAS*, *NRAS*, and *BRAF* genes.

Several randomized clinical trials (RCTs) and cohort studies have suggested differences in molecular makeup and survival outcomes between right- and left-sided CRC, and possibly different responses to targeted therapies [12,13,14,15,16]. However, current research efforts primarily focus on a binary classification of *KRAS*, *NRAS*, and *BRAF* genes as wildtype vs. mutant. This approach overlooks the potential differences of specific nucleotide changes within these three genes. As such, the present study aimed to examine the association between specific *KRAS*, *NRAS*, and *BRAF* hot-spot mutations and sidedness of primary CRC.

## 2. Materials and Methods

### 2.1. Study Cohort

This retrospective cohort study included 722 primary CRC patients seen at the University of Texas Medical Branch healthcare system (UTMB) from January 2016 through July 2023, all of whom had their tumors submitted for *KRAS*, *NRAS*, and *BRAF* mutational analysis. Patient demographics, clinical information, pathological features, and genetic data were abstracted and retrospectively assessed using the UTMB Molecular Diagnostic Lab database and UTMB electronic health record system (EPIC). Upon concluding the data collection phase, the dataset was de-identified to ensure patient confidentiality. This study was approved by the UTMB Institutional Review Board (IRB), Galveston, TX (IRB #: 02-089).

### 2.2. KRAS, NRAS, and BRAF Mutational Profiling

Mutational analysis was performed on a primary tumor where tissue was available from curative surgical resection or diagnostic biopsies. According to the manufacture protocol (Qiagen, Germantown, MD, USA), genomic DNA was extracted from formalin-fixed paraffin-embedded (FFPE) tissue obtained from available resections or biopsies using the QIAamp DNA FFPE Tissue Kit. Isolated genomic DNA was amplified using polymerase chain reaction (PCR) for segments of interest of *KRAS* gene covering codons (12, 13, and 61), *NRAS* gene covering codons (12, 13, and 61), and *BRAF* gene covering codon 600. The PCR protocol setting used included initial denaturation at 95 °C for 15 min, followed by 42 cycles of amplification at 95 °C for 20 s (denaturation), 53 °C for 30 s (annealing), and 72 °C for 20 s (elongation), with a final elongation step at 72 °C for 5 min. Prior to pyrosequencing, the quality of PCR products was checked using agarose gel electrophoresis against positive and negative controls. The mutations in *KRAS*, *NRAS*, and *BRAF* segments of interest were quantified in real time using pyrosequencing on the PyroMark Q24 System [17].

### 2.3. Study Measures

The outcome variable was primary CRC sidedness, categorized as right-sided (including tumors of the ileocecal valve, cecum, ascending colon, and hepatic flexure), transverse, left-sided (including tumors of the splenic flexure, descending colon, and sigmoid), or rectum.

Three primary predictor variables were assessed: *KRAS* mutation (classified as wildtype, p.Gly12Asp “G12D”, p.Gly12Val “G12V”, p.Gly12Cys “G12C”, Other p.Gly12 mutations, and p.Gly13Asp “G13D”), *NRAS* mutation (classified as wildtype, p.Gln61 mutations, other mutations, or unknown status), and *BRAF* mutation (dichotomized as wildtype or p.Val600Glu “V600E”). To account for potential confounding effects, we collected demographic and clinical data on each patient, including age at diagnosis, sex, race/ethnicity, familial risk of cancer, tobacco use, comorbidities, and DNA mismatch repair status. Detailed information on the study measures can be found in Table S1.

### 2.4. Statistical Analysis

Descriptive statistics were used to summarize the baseline characteristics of the study cohort. Categorical variables were presented as frequencies and proportions, while continuous variable was described using means with standard deviations (SD) and medians with interquartile ranges (IQR).

To evaluate the associations between specific mutations and the primary CRC sidedness (right colon, transverse colon, left colon, and rectum), we employed separate multivariable binary logistic regression models for each sidedness category. In each model, the outcome variable was defined as having primary CRC in the specific location of interest (e.g., left colon cancer) compared to all other locations (right, transverse, and rectum). This approach provides odds ratios that estimate the likelihood of developing cancer in the specific location compared to the combined risk of developing cancer elsewhere in the colon or rectum. All four regression models goodness-of-fit were assessed by the Hosmer−Lemeshow tests.

To account for the mutually exclusive nature of primary CRC sidedness categories, we conducted a sensitivity analysis using a multinomial logistic regression model (baseline-category logit). This model simultaneously analyzed primary CRC as a nominal variable with four categories (right-sided “reference category”, transverse, left-sided, rectum). A two-sided *p*-value < 0.05 was considered statistically significant. Data analyses and statistical modeling were performed using R software (version 4.3.1) and SAS software (SAS Institute Inc., v. 9.4, Cary, NC, USA).

## 3. Results

Table 1 and Table 2 and Figures S1–S7 illustrate *KRAS*, *NRAS*, and *BRAF* hot-spot mutations and patient characteristics stratified by primary CRC sidedness. A total of 722 primary CRC patients were analyzed. Of those, 220 (30.5%) had right-sided, 45 (6.2%) transverse, 249 (34.5%) left-sided, and 208 (28.8%) rectal tumors. Patients were, on average, 62.2 years old at diagnosis, with older ages noted in right-sided (64.7 years) and transverse tumors (65.7 years). Males comprised 66.5% of the patient population.

*KRAS* mutations were detected in 37.8% of cases, predominantly p.Gly12Asp (G12D) mutation. (13.2%). Right-sided primary CRC had the highest prevalence of mutated tumors (64%), followed by transverse (51.1%), rectal (43.2%), and then left-sided (39%) primary CRC. The distribution of *KRAS* mutations varied by sidedness, with 39.6% of p.Gly12Asp (G12D) mutation in right-sided tumors and 39.7% of p.Gly12Val (G12V) mutation in left-sided tumors. *KRAS* p.Gly12Cys (G12C) mutation was frequent in rectal tumors (52.4%), while p.Gly13Asp (G13D) mutation predominated in right-sided tumors (45.3%). Most *KRAS* codon 61 mutations occurred in rectal tumors (47.1%). *NRAS* mutations were found in 4.6% of cases, primarily p.Gln61 mutations (2.9%, with p.Gln61Lys (Q61K) mutation accounting for 1.7%). *BRAF* c.1799T>A (V600E) mutation was detected in 6.1% of cases, predominantly in right-sided tumors (70.5%). Among the 81 (11.2%) MMR-deficient tumors, the majority (*n* = 52, 64.2%) were located in the right colon.

**Figure 1:**
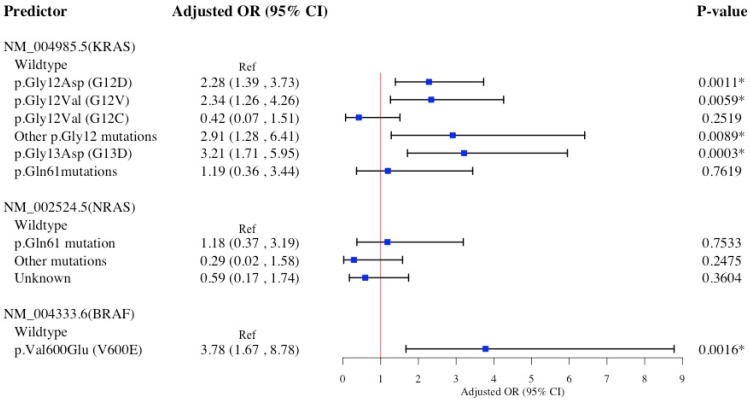
Multivariable logistic regression model for *KRAS*, *NRAS*, and *BRAF* hot-spot mutations association with primary right-colon cancer (*n* = 722). Multivariable multinomial regression model adjusted for age at diagnosis, sex, race/ethnicity, familial risk, tobacco use, comorbidities, DNA mismatch repair. Abbreviations: OR, odds ratio; CI, confidence interval; *BRAF*, v-raf murine sarcoma viral oncogene homolog B1; *NRAS*, neuroblastoma RAS viral oncogene homolog; *KRAS*, Kirsten rat sarcoma viral oncogene homolog. * Denotes statistical significance at the *p*-value < 0.05 level.

Figure 1 presents the association of specific *KRAS*, *NRAS*, and *BRAF* hot-spot mutations with right-sided CRC in a multivariable logistic model. *KRAS* p.Gly12Asp (G12D) mutation showed a significantly 128% increased likelihood of right-sided CRC compared to *KRAS* wildtype. Similarly, the *KRAS* p.Gly12Val (G12V) mutation, other p.Gly12 mutations, and the p.Gly13Asp (G13D) mutation were each associated with significantly increased likelihoods of right-sided CRC: 134%, 191%, and 221% higher, respectively. Likewise, *BRAF* p.Val600Glu (V600E) mutation, as opposed to *BRAF* wildtype, was associated with a significantly 278% higher likelihood of right-sided CRC.

**Figure 2:**
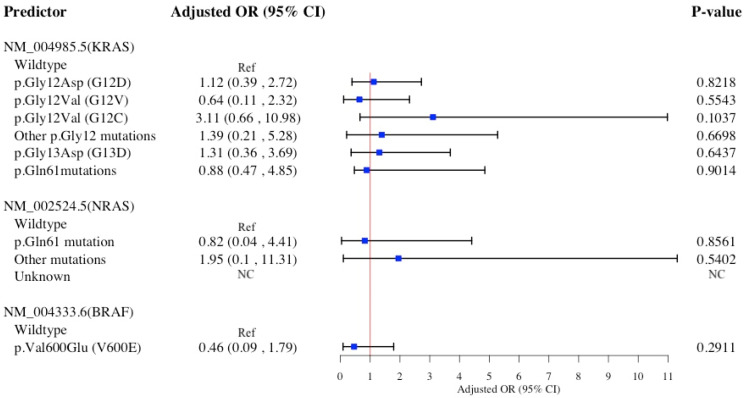
Multivariable logistic regression model for *KRAS*, *NRAS*, and *BRAF* hot-spot mutations association with primary Transverse colon cancer (*n* = 722). Multivariable regression models adjusted for age at diagnosis, sex, race/ethnicity, familial risk, tobacco use, comorbidities, DNA mismatch repair. Abbreviations: OR, odds ratio; CI, confidence interval; NC, not calculated; *BRAF*, v-raf murine sarcoma viral oncogene homolog B1; *NRAS*, neuroblastoma RAS viral oncogene homolog; *KRAS*, Kirsten rat sarcoma viral oncogene homolog.

Figure 2 illustrates the association of specific *KRAS*, *NRAS*, and *BRAF* hot-spot mutations with primary transverse colon cancer in a multivariable logistic model. No statistically significant associations were observed between these mutations and transverse colon cancer.

**Figure 3:**
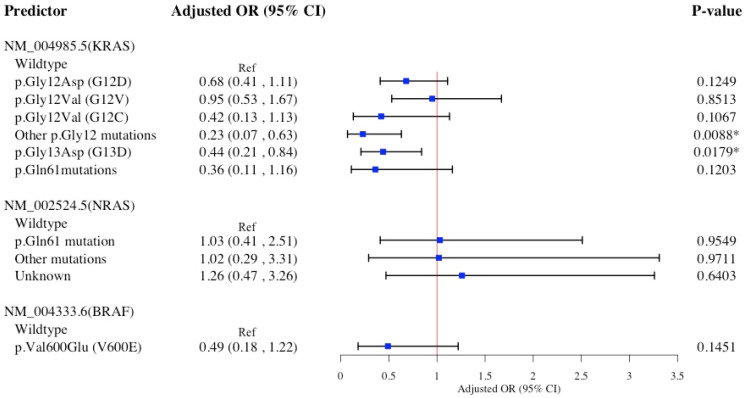
Multivariable logistic regression model for *KRAS*, *NRAS*, and *BRAF* hot-spot mutations association with primary Left colon cancer (*n* = 722). Multivariable regression models adjusted for age at diagnosis, sex, race/ethnicity, familial risk, tobacco use, comorbidities, DNA mismatch repair. Abbreviations: OR, odds ratio; CI, confidence interval; *BRAF*, v-raf murine sarcoma viral oncogene homolog B1; *NRAS*, neuroblastoma RAS viral oncogene homolog; *KRAS*, Kirsten rat sarcoma viral oncogene homolog. * Denotes statistical significance at the *p*-value < 0.05 level.

Figure 3 shows the association of specific *KRAS*, *NRAS*, and *BRAF* hot-spot mutations with primary left-sided CRC in a multivariable logistic model. Compared to *KRAS* wildtype, other p.Gly12 mutations and the p.Gly13Asp (G13D) mutation were significantly associated with reduced likelihood of left-sided CRC, 77% and 56% lower, respectively. No statistically significant associations were observed between any other *KRAS*, *NRAS*, or BRAF mutations and left-sided CRC.

**Figure 4:**
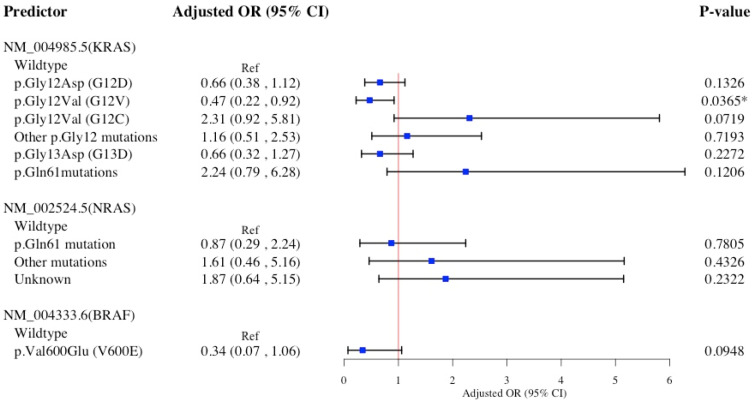
Multivariable logistic regression model for *KRAS*, *NRAS*, and *BRAF* hot-spot mutations association with primary Rectal cancer (*n* = 722). Multivariable multinomial regression model adjusted for age at diagnosis, sex, race/ethnicity, familial risk, tobacco use, comorbidities, DNA mismatch repair. Abbreviations: aOR, adjusted odds ratio; CI, confidence interval; *BRAF*, v-raf murine sarcoma viral oncogene homolog B1; *NRAS*, neuroblastoma RAS viral oncogene homolog; *KRAS*, Kirsten rat sarcoma viral oncogene homolog. * Denotes statistical significance at the *p*-value < 0.05 level.

Figure 4 displays the association of specific *KRAS*, *NRAS*, and *BRAF* hot-spot mutations with primary rectal cancer in a multivariable logistic model. *KRAS* p.Gly12Val (G12V) mutation, as opposed to *KRAS* wildtype, was associated with a significantly 53% lower likelihood of rectal cancer. No statistically significant associations were observed between any other *KRAS*, *NRAS*, or *BRAF* mutations and rectal cancer.

The Hosmer−Lemeshow test suggests all four multivariable logistic models presented in Figure 1, Figure 2, Figure 3 and Figure 4 accurately predict the data, with high *p*-values indicating no statistically significant differences between observed and predicted values (Table S2). Findings from sensitivity analyses using multinomial logistic models (Table S3) supported the associations between specific *KRAS* and *BRAF* hot-spot mutations and primary CRC sidedness, previously identified in (Figure 1, Figure 2, Figure 3 and Figure 4) binary logistic models.

## 4. Discussion

This retrospective cohort study set out to assess the association between *KRAS*, *NRAS*, and *BRAF* hot-spot mutations and primary CRC sidedness. Specific mutations in *KRAS* (p.Gly12Asp, p.Gly12Val, and p.Gly13Asp) and *BRAF* (p.Val600Glu) were associated with increased likelihood of right-sided CRC. Conversely, *KRAS* p.Gly12Val and p.Gly13Asp mutations were associated with decreased likelihood of rectal cancer and left-sided CRC, respectively. There was no evidence of a significant association between *NRAS* hot-spot mutations and CRC sidedness.

Similar to previous research, the mutation frequencies of *KRAS* and *BRAF* exhibit notable disparities contingent upon CRC sidedness, with higher mutation frequency in right-sided CRC [18,19,20,21]. These mutational events may stem from diverse etiological factors, including DNA modifications, defective DNA repair mechanisms, or exposure to mutagens, collectively promoting tumor cell proliferation and survival [22,23,24].

Our study’s results are consistent with existing research. For instance, a meta-analysis of 25 studies has demonstrated a significant link between *BRAF* (p.Val600Glu) mutation and CRC sidedness [25]. Additionally, a large-scale U.S. study using the National Cancer Institute’s SEER program identified a link between *KRAS* hot-spot mutations and CRC sidedness [26]. Supporting these findings, a study of 2413 biospecimens confirmed that CRC sidedness is associated with both *KRAS* and *BRAF* hot-spot mutations [20].

CRC manifests molecular heterogeneity, characterized by a spectrum of genetic and epigenetic alterations affecting oncogenes and tumor suppressor genes [27]. At the core of CRC tumorigenesis lies the constitutive activation of the RAS/RAF/MEK/ERK pathway, modulated by gene mutations and regulatory actions of RAS and BRAF [2]. Our findings, in conjunction with prior research, emphasize the molecular heterogeneity and diverse mutational processes of primary CRC. While *KRAS*, *NRAS*, and *BRAF* hot-spot mutations are very important drivers of tumorigenesis and tumor progression, it is important to note that they are not the only intrinsic features of colonic tissues dictating its consequences for tumorigenesis. It is biologically plausible that the intestinal microenvironment, epigenetic modifications, and tumor−stroma interactions are involved in shaping tumorigenesis across diverse tumor locations.

The study’s key strengths lie in the utilization of high-quality, real-world clinical data accurately reflecting contemporary clinical practice, and the employment of consistent pyrosequencing-based tumor genotyping to ensure internal study validity. The relatively large sample size enhances the reliability and generalizability of our findings to the broader CRC patient population. *KRAS*, *NRAS*, and *BRAF* hot-spot mutations statistical analysis at both nucleotide and protein changes offers a more nuanced perspective and granular picture underlying mutational processes driving tumor formation in different colon regions.

However, our study has several limitations. First, its single-institution design limits generalizability. Second, the retrospective nature of the study may introduce selection bias. Third, the pyrosequencing method used only detects mutations in *KRAS* and *NRAS* codons 12, 13, and 61, and *BRAF* codon 600. Fourth, pyrosequencing has a sensitivity threshold of approximately 10% mutant alleles. Finally, while we adjusted for potential confounders in our multivariable regression models, the impact of unmeasured factors, such as other genetic mutations, and residual confounding cannot be completely ruled out.

## 5. Conclusions

In summary, this study contributes to the existing knowledge on the association between *KRAS*, *NRAS*, and *BRAF* hot-spot mutations and primary CRC sidedness. Distribution of specific *KRAS* and *BRAF* hot-spot mutations significantly varied by sidedness. Hot-spot mutations were most frequently found in right-sided tumors, with transverse tumors coming next in prevalence, followed by rectal and then left-sided tumors. In multivariable regression models, specific mutations in *KRAS* and *BRAF*, which lead to aberrant activation of the RAS/RAF/MEK/ERK signaling pathway, were associated with CRC sidedness. *KRAS* (p.Gly12Asp, p.Gly12Val, and p.Gly13Asp) and *BRAF* (p.Val600Glu) mutations were associated with increased likelihood of right-sided CRC. Conversely, *KRAS* p.Gly12Val and p.Gly13Asp mutations were associated with decreased likelihood of rectal cancer and left-sided CRC respectively. These findings underscore the distinct *KRAS* and *BRAF* hot-spot mutations for the different anatomical locations of CRC, suggesting distinct tumorigenesis and mutational processes across tumor sidedness. Thus, prognostic stratification contingent on primary CRC sidedness along with the *KRAS* and *BRAF* hot-spot mutational status is a reasonable approach. Further studies are needed to substantiate these findings.

## Figures and Tables

**Table 1:** Distribution of protein and nucleotide changes in *KRAS*, *NRAS,* and *BRAF* hot-spot mutations according to primary CRC sidedness (*n* = 722).

Proto-Oncogenes Hot-Spot Mutations		Overall *n* (%)	Primary CRC Sidedness *			
Protein Change	Nucleotide Change		Right 220 (30.5)	Transverse 45 (6.2)	Left 249 (34.5)	Rectum 208 (28.8)
NM_004985.5(*KRAS*) mutations						
*KRAS* wildtype	N/A	449 (62.2)	117 (26.1)	27 (6.0)	173 (38.5)	132 (29.4)
*KRAS* codon 12						
p.Gly12Asp (G12D)	c.35G>A	96 (13.2)	38 (39.6)	6 (6.3)	30 (31.2)	22 (22.9)
p.Gly12Val (G12V)	c.35G>T	58 (8.0)	22 (37.9)	2 (3.4)	23 (39.7)	11 (19.0)
p.Gly12Cys (G12C)	c.34G>T	21 (2.9)	2 (9.5)	3 (14.3)	5 (23.8)	11 (52.4)
p.Gly12Ser (G12S)	c.34G>A	17 (2.4)	4 (23.5)	1 (5.9)	3 (17.7)	9 (52.9)
p.Gly12Ala (G12A)	c.35G>C	8 (1.1)	5 (62.5)	1 (12.5)	1 (12.5)	1 (12.5)
p.Gly12Arg (G12R)	c.34G>C	4 (0.5)	3 (75.0)	0 (0.0)	0 (0.0)	1 (25.0)
*KRAS* codon 13						
p.Gly13Asp (G13D)	c.38G>A	53 (7.3)	24 (45.3)	4 (7.6)	12 (22.6)	13 (24.5)
*KRAS* codon 61						
p.Gln61His (Q61H)	c.183A>T	5 (0.7)	1 (20.0)	1 (20.0)	1 (20.0)	2 (40.0)
	c.183A>C	5 (0.7)	3 (60.0)	0 (0.0)	0 (0.0)	2 (40.0)
p.Gln61Leu (Q61L)	c.182A>T	4 (0.5)	1 (25.0)	0 (0.0)	2 (50.0)	1 (25.0)
p.Gln61Arg (Q61R)	c.182A>G	3 (0.4)	0 (0.0)	0 (0.0)	0 (0.0)	3 (100.0)
p.Gln61Glu (Q61E)	c.181C>G	1 (0.1)	0 (0.0)	0 (0.0)	0 (0.0)	1 (100.0)
NM_002524.5(*NRAS*) mutations						
*NRAS* wildtype	N/A	668 (92.5)	208 (31.1)	43 (6.5)	227 (34.0)	190 (28.4)
*NRAS* codon 12						
p.Gly12Asp (G12D)	c.35G>A	5 (0.7)	1 (20.0)	1 (20.0)	1 (20.0)	2 (40.0)
p.Gly12Val (G12V)	c.35G>T	3 (0.4)	0 (0.0)	0 (0.0)	1 (33.3)	2 (66.7)
p.Gly12Cys (G12C)	c.34G>T	1 (0.1)	0 (0.0)	0 (0.0)	1 (100.0)	0 (0.0)
p.Gly12Ser (G12S)	c.34G>A	1 (0.1)	0 (0.0)	0 (0.0)	1 (100.0)	0 (0.0)
*NRAS* codon 13						
p.Gly13Val (G13V)	c.38G>T	2 (0.3)	0 (0.0)	0 (0.0)	1 (50.0)	1 (50.0)
*NRAS* codon 61						
p.Gln61Lys (Q61K)	c.181C>A	12 (1.7)	4 (33.3)	1 (8.3)	5 (41.7)	2 (16.7)
p.Gln61Arg (Q61R)	c.182A>G	6 (0.8)	1 (16.7)	0 (0.0)	3 (50.0)	2 (33.3)
p.Gln61His (Q61H)	c.183A>T	2 (0.3)	0 (0.0)	0 (0.0)	1 (50.0)	1 (50.0)
p.Gln61Leu (Q61L)	c.182A>T	1 (0.1)	0 (0.0)	0 (0.0)	1 (100.0)	0 (0.0)
Unknown *NRAS*	N/A	21 (2.9)	6 (28.6)	0 (0.0)	8 (38.1)	7 (33.3)
NM_004333.6(*BRAF*) *V600* mutations						
*BRAF* V600 wildtype	N/A	678 (93.9)	189 (27.9)	42 (6.2)	242 (35.7)	205 (30.2)
p.Val600Glu (V600E)	c.1799T>A	44 (6.1)	31 (70.5)	3 (6.8)	7 (15.9)	3 (6.8)

Data are presented as count (*n*) and percentage (%). Abbreviations: *BRAF*, v-raf murine sarcoma viral oncogene homolog B1; *NRAS*, neuroblastoma RAS viral oncogene homolog; *KRAS*, Kirsten rat sarcoma viral oncogene homolog. * Left colon forms splenic flexure, descending colon, sigmoid colon. Right colon forms ileocecal valve, hepatic flexure, cecum, ascending colon.

**Table 2:** Patient characteristics according to primary CRC sidedness (*n* = 722).

Characteristic	Overall *n* (%)	Primary CRC Sidedness			
		Right 220 (30.5)	Transverse 45 (6.2)	Left 249 (34.5)	Rectum 208 (28.8)
Age at diagnosis					
Mean ± SD	62.2 ± 12.6	64.7 ± 12.6	65.7 ± 12.6	61.3 ± 12.4	59.8 ± 12.3
Median, IQR	62, 16	66, 16	65, 20	61, 16	61, 15
Sex					
Male	480 (66.5)	130 (27.1)	31 (6.4)	175 (36.5)	144 (30.0)
Female	242 (33.5)	90 (37.2)	14 (5.8)	74 (30.6)	64 (26.4)
Race/ethnicity					
White	398 (55.1)	118 (29.6)	25 (6.3)	148 (37.2)	107 (26.9)
Hispanic	160 (22.2)	48 (30.0)	10 (6.3)	49 (30.6)	53 (33.1)
Black	150 (20.8)	52 (34.7)	8 (5.3)	45 (30.0)	45 (30.0)
Other	14 (1.9)	2 (14.3)	2 (14.3)	7 (50.0)	3 (21.4)
NM_004985.5(*KRAS*) mutations					
Wildtype	449 (62.2)	117 (26.1)	27 (6.0)	173 (38.5)	132 (29.4)
p.Gly12Asp (G12D)	95 (13.2)	38 (40.0)	6 (6.3)	29 (30.5)	22 (23.2)
p.Gly12Val (G12V)	58 (8.0)	22 (37.9)	2 (3.4)	23 (39.7)	11 (19.0)
p.Gly12Val (G12C)	21 (2.9)	2 (9.5)	3 (14.3)	5 (23.8)	11 (52.4)
Other p.Gly12 mutations	29 (4.0)	12 (41.4)	2 (6.9)	4 (13.8)	11 (37.9)
p.Gly13Asp (G13D)	53 (7.3)	24 (45.3)	4 (7.6)	12 (22.6)	13 (24.5)
p.Gln61mutations	17 (2.4)	5 (29.4)	1 (5.9)	3 (17.6)	8 (47.1)
NM_002524.5(*NRAS*) mutations					
Wildtype	668 (92.5)	208 (31.1)	43 (6.5)	227 (34.0)	190 (28.4)
p.Gln61 mutations	21 (2.9)	5 (23.8)	1 (4.7)	9 (42.9)	6 (28.6)
Other mutations	12 (1.7)	1 (8.3)	1 (8.3)	5 (41.7)	5 (41.7)
Unknown	21 (2.9)	6 (28.6)	0 (0.0)	8 (38.1)	7 (33.3)
NM_004333.6(*BRAF*) mutations					
Wildtype	678 (93.9)	189 (27.9)	42 (6.2)	242 (35.7)	205 (30.2)
p.Val600Glu (V600E)	44 (6.1)	31 (70.5)	3 (6.8)	7 (15.9)	3 (6.8)
DNA mismatch repair					
MMR-proficient	641 (88.8)	168 (26.2)	35 (5.5)	236 (36.8)	202 (31.5)
MMR-deficient	81 (11.2)	52 (64.2)	10 (12.3)	13 (16.0)	6 (7.5)
Familial risk					
No	417 (57.8)	123 (29.5)	27 (6.4)	145 (34.8)	122 (29.3)
Yes	305 (42.2)	97 (31.8)	18 (5.9)	104 (34.1)	86 (28.2)
Tobacco use					
No	269 (37.3)	93 (34.6)	16 (5.9)	86 (32.0)	74 (27.5)
Yes	453 (62.7)	127 (28.0)	29 (6.4)	163 (36.0)	134 (29.6)
Comorbidities					
0	131 (18.1)	30 (22.9)	6 (4.6)	44 (33.6)	51 (38.9)
1–2	319 (44.2)	94 (29.5)	16 (5.0)	118 (37.0)	91 (28.5)
3 or more	272 (37.7)	96 (35.3)	23 (8.4)	87 (32.0)	66 (24.3)

Data are presented as count (*n*) and percentage (%) unless otherwise indicated. Other race/ethnicity includes Asian (*n* = 11) and American Indian/Alaska Native (*n* = 3). Other *KRAS* and *NRAS* mutations described in Table 1. Abbreviations: SD; standard deviation; IQR, interquartile range; *BRAF*, v-raf murine sarcoma viral oncogene homolog B1; *NRAS*, neuroblastoma RAS viral oncogene homolog; *KRAS*, Kirsten rat sarcoma viral oncogene homolog. Other *KRAS* p.Gly12 mutations include p.Gly12Ser (G12S), p.Gly12Ala (G12A), and p.Gly12Arg (G12R). The *KRAS* p.Gln61mutations include p.Gln61His (Q61H), p.Gln61Leu (Q61L), p.Gln61Arg (Q61R), and p.Gln61Glu (Q61E). The *NRAS* p.Gln61 mutations include p.Gln61Lys (Q61K), p.Gln61Arg (Q61R), p.Gln61His (Q61H), and p.Gln61Leu (Q61L). Other *NRAS* mutations include p.Gly12Asp (G12D), p.Gly12Val (G12V), p.Gly12Cys (G12C), p.Gly12Ser (G12S), and p.Gly13Val (G13V).

## Data Availability

The data presented in this study are available upon reasonable request from the corresponding author. The data are not publicly available due to patient confidentiality restrictions.

## Supplementary Materials

The online version contains supplementary material available online: https://www.mdpi.com/article/10.3390/diagnostics15020142/s1. Figure S1. Overall distribution of *KRAS*, *NRAS*, and *BRAF* hot-spot mutations according to primary CRC sidedness. Figure S2. Overall distribution of *KRAS* mutations in primary CRC. Figure S3. *KRAS* mutations according to primary CRC sidedness. Figure S4. Overall distribution of *NRAS* mutations in primary CRC. Figure S5. *NRAS* mutations according to primary CRC sidedness. Figure S6. Overall distribution of *BRAF* mutations in primary CRC. Figure S7. *BRAF* mutations according to primary CRC sidedness. Table S1. Study’s variables and attributes. Table S2. The Hosmer-Lemeshow goodness-of-fit tests for multivariable logistic models predicting CRC sidedness. Table S3. Multivariable multinomial baseline-category logistic regression for *KRAS*, *NRAS*, and *BRAF* hot-spot mutations association with primary CRC sidedness.
