# Supplementary material for: KRAS, NRAS, and BRAF Hot-Spot Mutations in Relation to Sidedness of Primary Colorectal Cancer: A Retrospective Cohort Study
Source: Diagnostics (Basel). 2025 Jan 9;15(2):142. doi: 10.3390/diagnostics15020142 (PMC11763696; doi:10.3390/diagnostics15020142)
Supplement: Supplementary file 1 [file diagnostics-15-00142-s001.zip › diagnostics-3340028-supplementary.pdf]

Supplementary Materials

KRAS, *NRAS*, and *BRAF* Hot-Spot Mutations in Relation to Sidedness of Primary Colorectal Cancer: A Retrospective Cohort Study

Supplemental Materials Titles

Figure S1. Overall distribution of *KRAS*, *NRAS* and *BRAF* hot-spot mutations according to primary CRC sidedness (n=722).
Figure S2. Overall distribution of *KRAS* mutations in primary CRC (n=722).
Figure S3. *KRAS* mutations according to primary CRC sidedness (n=722).
Figure S4. Overall distribution of *NRAS* mutations in primary CRC (n=722).
Figure S5. *NRAS* mutations according to primary CRC sidedness (n=722).
Figure S6. Overall distribution of *BRAF* mutations in primary CRC (n=722).
Figure S7. *BRAF* mutations according to primary CRC sidedness (n=722).
Table S1. Study's variables and attributes.
Table S2. The Hosmer-Lemeshow goodness-of-fit tests for multivariable logistic models predicting CRC sidedness.
Table S3. Multivariable multinomial baseline-category logistic regression for *KRAS, NRAS*, and *BRAF* hot-spot mutations association with primary CRC sidedness (n=722).

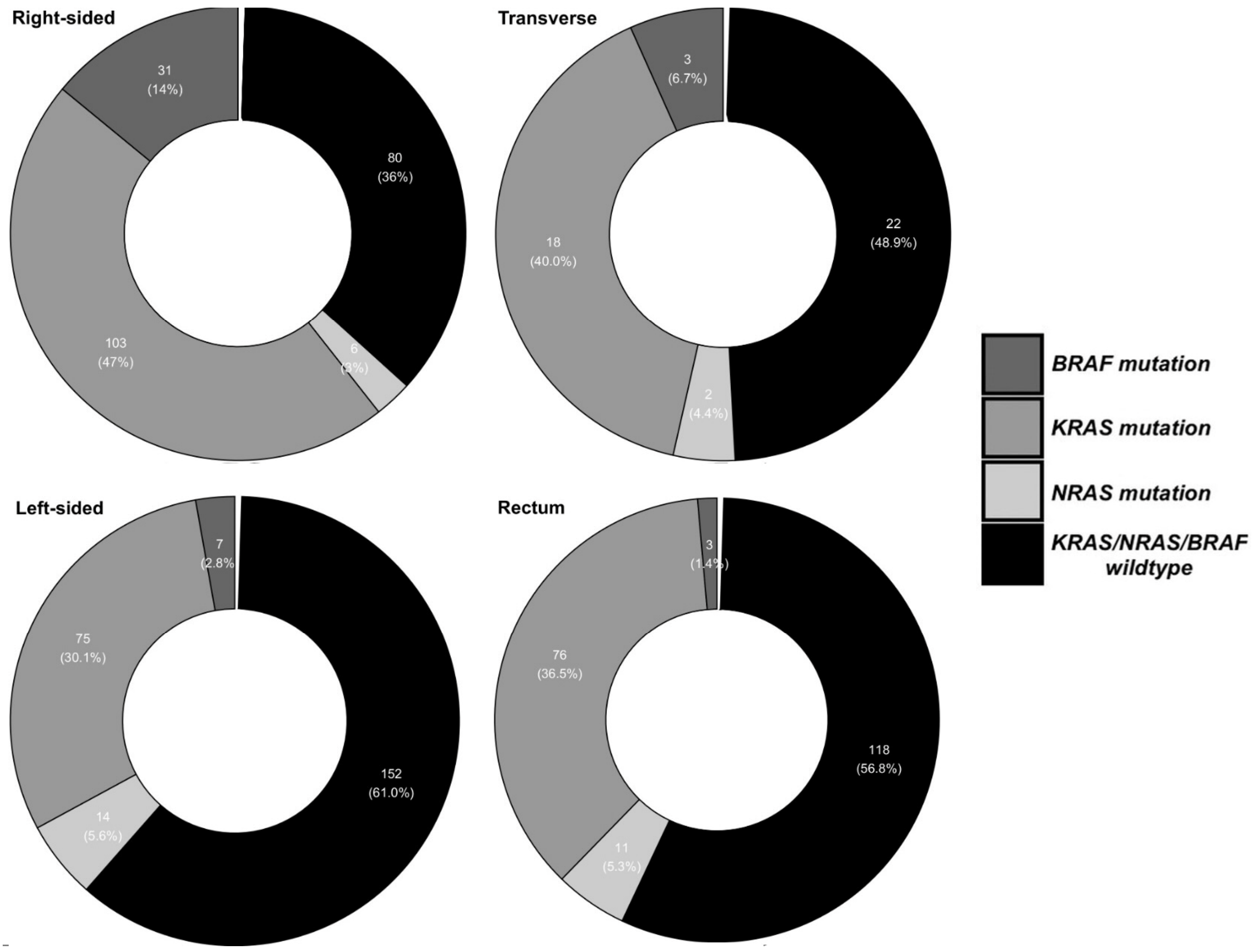

Figure S1. Overall distribution of *KRAS*, *NRAS* and *BRAF* hot-spot mutations according to primary CRC sidedness (n=722).

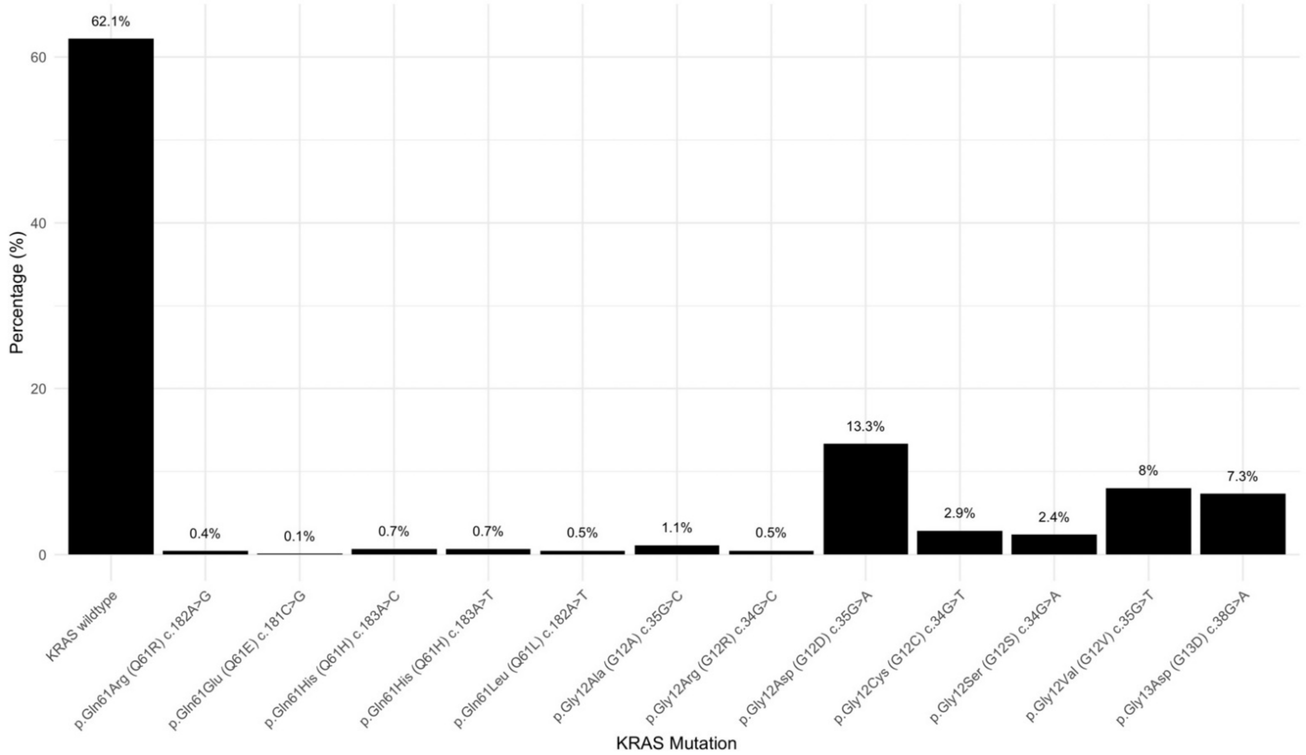

Figure S2. Overall distribution of *KRAS* mutations in primary CRC (n=722).

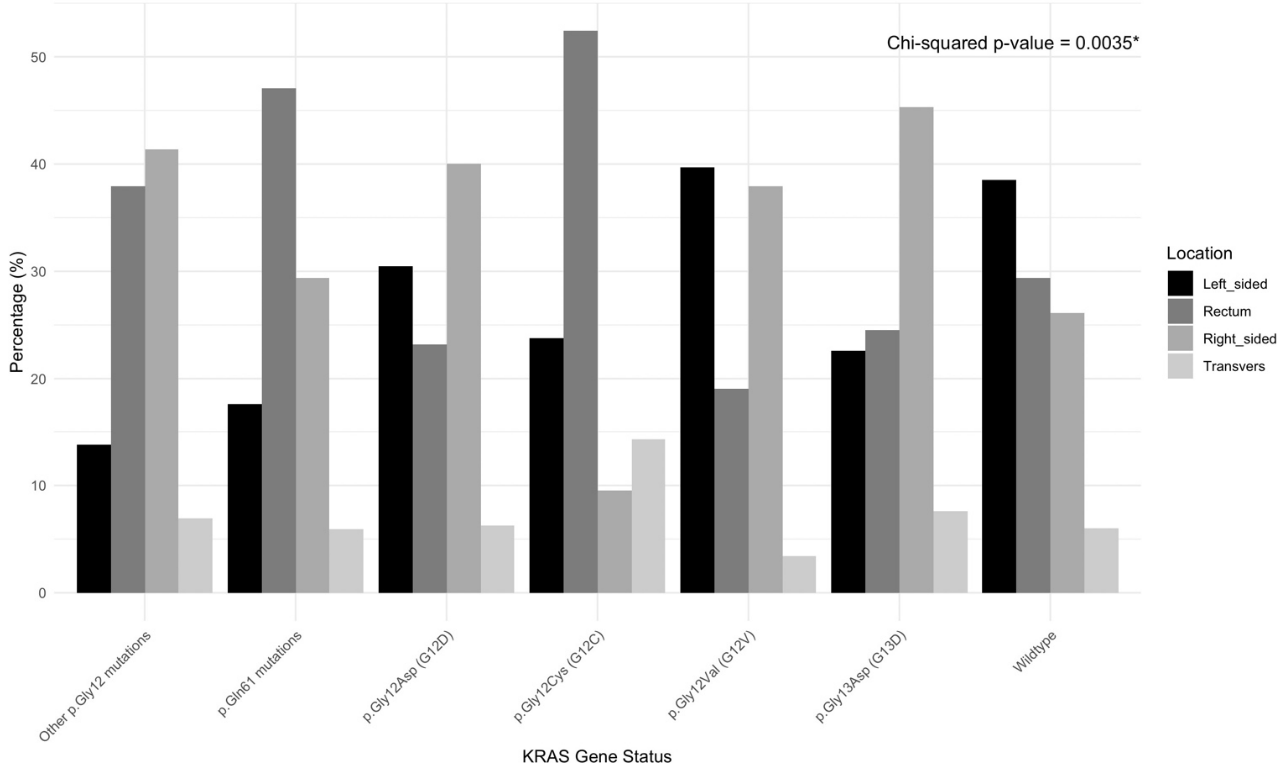

Figure S3. *KRAS* mutations according to primary CRC sidedness (n=722).

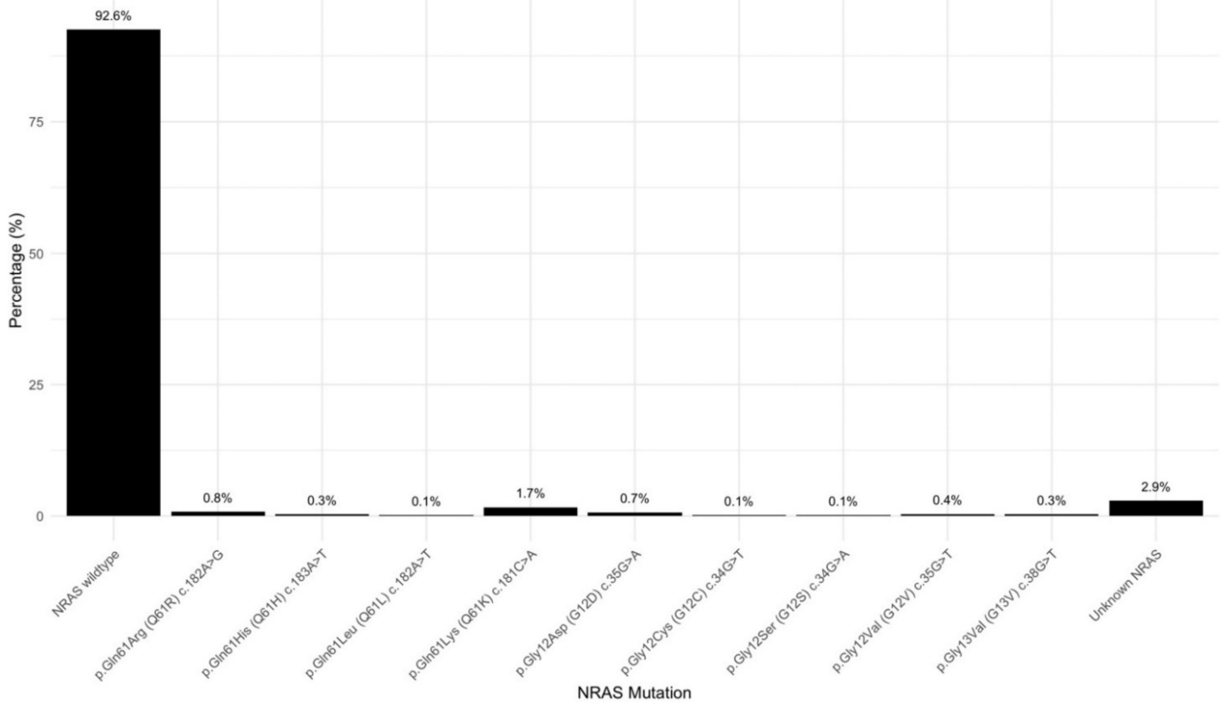

Figure S4. Overall distribution of *NRAS* mutations in primary CRC (n=722).

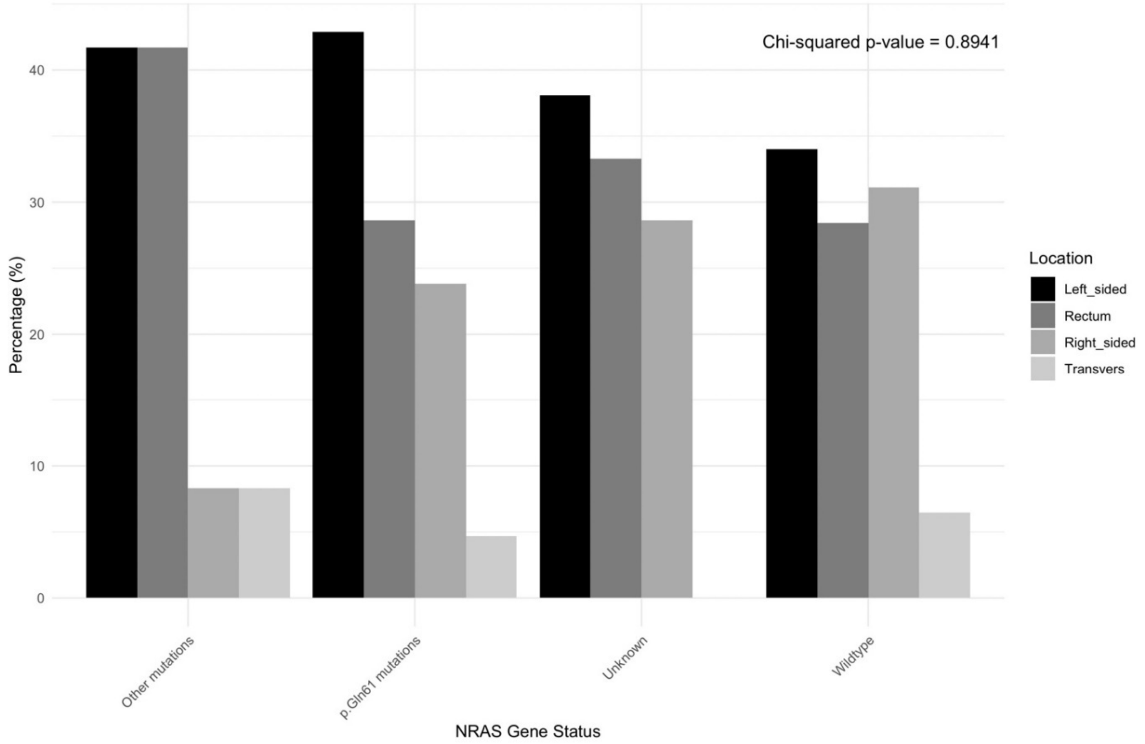

Figure S5. *NRAS* mutations according to primary CRC sidedness (n=722).

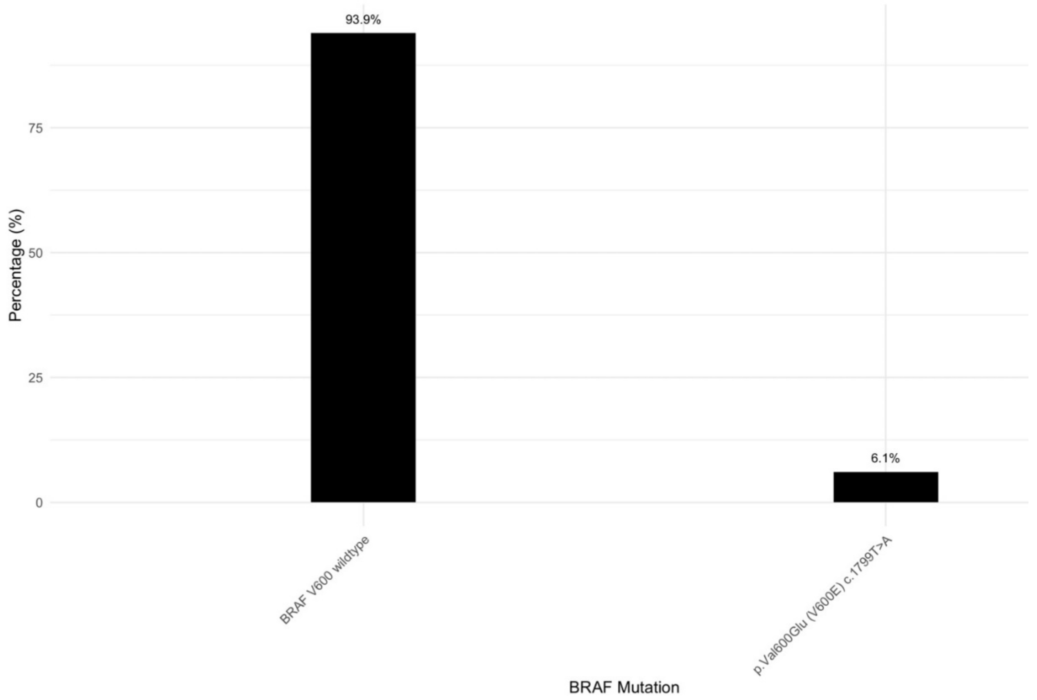

Figure S6. Overall distribution of *BRAF* mutations in primary CRC (n=722).

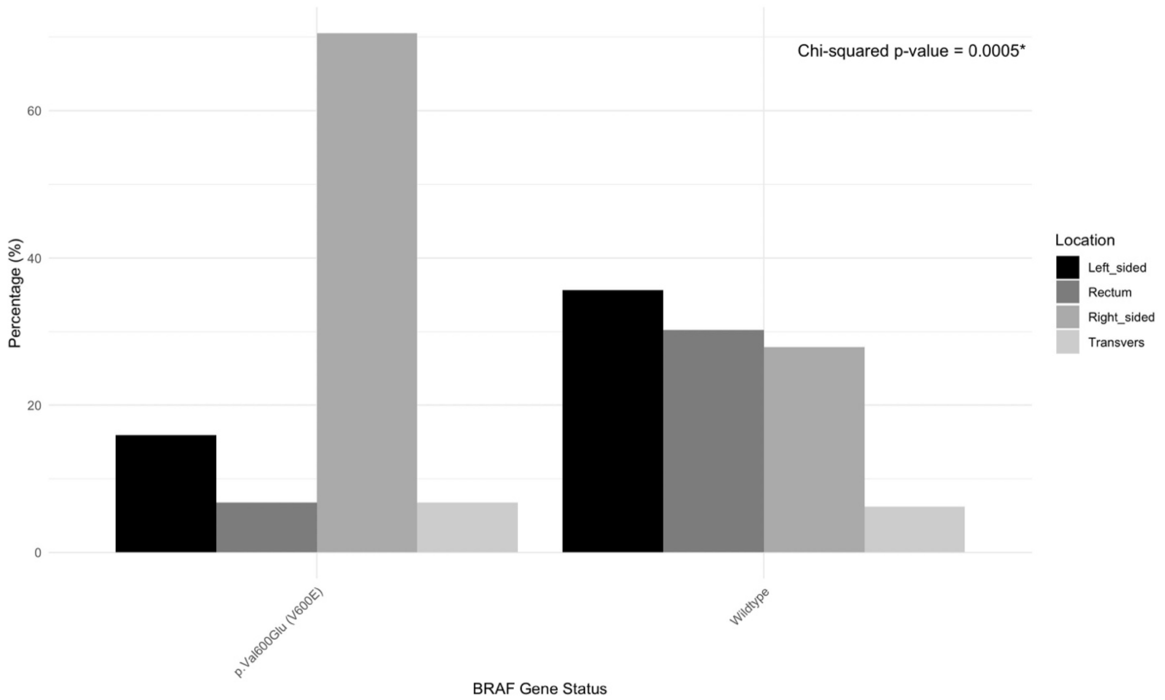

Figure S7. *BRAF* mutations according to primary CRC sidedness (n=722).

Table S1. Study's variables and attributes.

Variable	Description	Values
Primary CRC sidedness	Primary tumor anatomical location. Right colon included Ileocecal valve, cecum, ascending colon, hepatic flexure. Left colon included splenic flexure, descending colon, and sigmoid.	Right colon<br>Transvers colon<br>Left colon<br>Rectum
Age at diagnosis	Patient's age in years at initial CRC diagnosis.	Numbers
Sex	Patient's biological sex.	Male<br>Female
Race/ethnicity	Self-identified race/ethnicity group.	White<br>Hispanic<br>Black<br>Other
NM_004985.5(*KRAS*) mutations	Kirsten rat sarcoma viral oncogene homolog gene. Mutation in *KRAS* genomic regions; exon 2 (codon 12 and 13), exon 3 (codon 61). Other p.Gly12 mutations include p.Gly12Ser (G12S), p.Gly12Ala (G12A) and p.Gly12Arg (G12R). The p.Gln61mutations include p.Gln61His (Q61H), p.Gln61Leu (Q61L), p.Gln61Arg (Q61R) and p.Gln61Glu (Q61E).	Wildtype<br>p.Gly12Asp (G12D)<br>p.Gly12Val (G12V)<br>p.Gly12Val (G12C)<br>Other p.Gly12 mutations<br>p.Gly13Asp (G13D)<br>p.Gln61mutations
NM_002524.5(*NRAS*) mutations	Neuroblastoma RAS viral (V-ras) oncogene homolog. Mutation in *NRAS* genomic regions; exon 2 (codon 12 and 13), exon 3 (codon 61). p.Gln61 mutations include p.Gln61Lys (Q61K), p.Gln61Arg (Q61R), p.Gln61His (Q61H) and p.Gln61Leu (Q61L). Other mutations include p.Gly12Asp (G12D), p.Gly12Val (G12V), p.Gly12Cys (G12C), p.Gly12Ser (G12S) and p.Gly13Val (G13V).	Wildtype<br>p.Gln61 mutations<br>Other mutations<br>Unknown
NM_004333.6(*BRAF*) mutations	v-raf murine sarcoma viral oncogene homolog B1. *BRAF* V600E mutation.	Wildtype<br>p.Val600Glu (V600E)
DNA mismatch repair	Mismatch Repair (MMR). Microsatellite (genetic) instability in short nucleotide repeats. Expression of protein products of *MSH2, MSH6, MLH1* and *PMS2* genes by immunohistochemistry (IHC) assays.	MMR-proficient<br>MMR-deficient

Familial risk	Have relatives who have had CRC, polyp or any other types of cancer or gland-like growths developed on the mucous membrane that lines the large intestine.	No<br>Yes
Tobacco use	Self-reported use of tobacco product.	No<br>Yes
Comorbidities	Number of highly prevalent conditions that may influence cancer management alone or in combination with another condition Including other cancer, other metastatic cancer, CVD, CPD, CDH, MI, PVD, HTN, DM, peptic ulcer, rheumatological disease, dementia, hemiplegia and paraplegia, liver disease and renal disease.	0<br>1 - 2<br>3 or more

Table S2. The Hosmer-Lemeshow goodness-of-fit tests for multivariable logistic models predicting CRC sidedness.

**Logistic model**	**Statistic**	**degree of freedom**	**P-value**
Right colon cancer	4.10646	9	0.90427
Transvers colon cancer	6.20732	8	0.62402
Left colon cancer	5.83887	8	0.66527
Rectal cancer	10.4001	9	0.31908

Table S3. Multivariable multinomial baseline-category logistic regression for *KRAS, NRAS*, and *BRAF* hot-spot mutations association with primary CRC sidedness (n=722).

Predictor	Primary CRC sidedness					
	Right vs. Transverse		Right vs. Left		Right vs. Rectum	
	aOR (95% CI)	P-value	aOR (95% CI)	P-value	aOR (95% CI)	P-value
**NM_004985.5*(KRAS)***						
• Wildtype	Ref		Ref		Ref	
• p.Gly12Asp (G12D)	0.65 (0.24 , 1.75)	0.3895	0.43 (0.24 , 0.76)	**0.0039***	0.41 (0.22 , 0.76)	**0.0047***
• p.Gly12Val (G12V)	0.36 (0.08 , 1.71)	0.1996	0.53 (0.27 , 1.02)	0.0582	0.31 (0.14 , 0.69)	**0.0042***
• p.Gly12Val (G12C)	5.90 (0.88 , 39.66)	0.0678	1.19 (0.22 , 6.31)	0.8481	3.35 (0.71 , 15.82)	0.1270
• Other p.Gly12 mutations	0.66 (0.13, 3.29)	0.6157	0.16 (0.05 , 0.52)	**0.0024***	0.51 (0.21 , 1.25)	0.1426
• p.Gly13Asp (G13D)	0.63 (0.19 , 2.02)	0.4344	0.25 (0.12 , 0.54)	**0.0004***	0.32 (0.15, 0.68)	**0.0038***
• p.Gln61mutations	0.77 (0.08 , 7.05)	0.8145	0.43 (0.09 , 1.89)	0.2621	1.43 (0.42 , 4.85)	0.5620
**NM_002524.5*(NRAS)***						
• Wildtype	Ref		Ref		Ref	
• p.Gln61 mutation	0.74 (0.08 , 6.82)	0.7913	0.89 (0.28 , 2.79)	0.8446	0.79 (0.23 , 2.77)	0.7165
• Other mutations	5.32 (0.31 , 92.11)	0.2503	2.86 (0.32 , 25.41)	0.3451	3.81 (0.43 , 33.65)	0.2296
• Unknown	NC	NC	1.94 (0.53 , 7.08)	0.3144	2.64 (0.67 , 10.49)	0.1671
**NM_004333.6 *(BRAF)***						
• Wildtype	Ref		Ref		Ref	
• p.Val600Glu (V600E)	0.30 (0.07 , 1.29)	0.1071	0.28 (0.10 , 0.77)	**0.0131***	0.18 (0.05 , 0.69)	**0.0125***

Multivariable multinomial baseline logistic regression model adjusted for age at diagnosis, sex, race/ethnicity, familial risk, tobacco use, comorbidities, DNA mismatch repair. Abbreviations: aOR, adjusted odds ratio; CI, confidence interval; NC, not calculated; *BRAF*, v-raf murine sarcoma viral oncogene homolog B1; *NRAS*, neuroblastoma RAS viral oncogene homolog; *KRAS*, Kirsten rat sarcoma viral oncogene homolog. Other p.Gly12 mutations include p.Gly12Ser (G12S), p.Gly12Ala (G12A) and p.Gly12Arg (G12R). The p.Gln61mutations include p.Gln61His (Q61H), p.Gln61Leu (Q61L), p.Gln61Arg (Q61R) and p.Gln61Glu (Q61E). * Denotes statistical significance at the P-value < 0.05 level.
